# Comparison of the Effects of Ultrasound-Guided Subacromial Injection Versus Subacromial Injection and Suprascapular Nerve Block on Pain, Function, and Sleep Quality in Rotator Cuff Lesions

**DOI:** 10.3390/jcm13237258

**Published:** 2024-11-29

**Authors:** Mustafa Haciomeroglu, Suna Akin Takmaz, Azize Serce, Yilmaz Karaduman, Hulya Basar

**Affiliations:** 1 Ercis Sehit Ridvan Cevik State Hospital, 65400 Ercis-Van, Turkey; 2 Ankara Training and Research Hospital, 06200 Ankara, Turkey; satakmaz@gmail.com (S.A.T.); ymzkaraduman@hotmail.com (Y.K.); hulya_basar@yahoo.com (H.B.); 3 Canakkale Mehmet Akif Ersoy State Hospital, 17100 Canakkale, Turkey; azizedc37@hotmail.com

**Keywords:** shoulder pain, rotator cuff lesions, suprascapular nerve block, subacromial injection

## Abstract

**Background/Objectives:** Rotator cuff lesions are common causes of shoulder pain. When not treated effectively, the functional loss associated with pain affects the quality of life and brings about psychosocial issues. In this study, prospective observational comparison of the effects of ultrasonography (USG) guided subacromial injection (SAI) versus subacromial injection combined with suprascapular nerve block (SSNB) on pain, functionality and sleep quality in the treatment of shoulder pain unresponsive to conservative treatments due to rotator cuff lesions is made. **Methods:** The data of 25 patients in both groups were compared prospectively. Patients were evaluated after 30 min, 1 week, 2 weeks, 1 month, and 3 months. Pain levels were measured with VAS, shoulder functions with SPADI and sleep quality with PSQI. Analgesic consumption and satisfaction were also recorded. **Results:** Both treatment groups effectively reduced pain at rest over the 3-month follow-up period. However, the SAI group did not achieve the targeted level of analgesia for pain control during movement. In comparison, the SAI + SSNB group demonstrated significantly superior outcomes, with lower VAS scores both at rest and during motion, as well as improved SPADI and PSQI scores. Additionally, analgesic consumption was significantly reduced in the SAI + SSNB group. No side effects or complications were observed during the treatment applications or the follow-up period. **Conclusions:** Pain control, shoulder functionality, sleep quality, and patient satisfaction were found to be higher in patients treated with SAI + SSNB in the short-to-medium term in the treatment of shoulder pain due to rotator cuff lesions, in addition to lower analgesic consumption.

## 1. Introduction

The prevalence of shoulder pain ranges from 7% to 25% [1]. One of the most common causes is rotator cuff lesions (RCL). Among patients with RCL, 30% are unable to perform simple daily tasks, and almost 50% require hospitalization due to chronic shoulder pain. When not treated effectively, the functional loss associated with pain affects the quality of life and brings about psychosocial issues. Magnetic resonance imaging (MRI) and ultrasound are highly appreciated in the diagnosis of RCL. MRI and ultrasound have high resolution for articular cartilage, tendons, soft tissues, and ligaments, and they provide clear imaging features of the scanned area [2]. A meta-analysis revealed that the diagnostic sensitivity and specificity of ultrasound and MRI for rotator cuff tears have exceeded 90%, suggesting a promising diagnostic efficiency of imaging approaches for rotator cuff tears [3]. The aim of treatment is to alleviate pain, eliminate functional limitations, and facilitate a patient’s expedient reintegration into social and professional activities. A variety of conservative treatment modalities are used for this purpose, including rest, analgesic medications, and physical therapy techniques. However, conservative treatments are not always efficacious in providing adequate pain control, and some patients are unable to continue treatment due to the occurrence of adverse effects. In recent years, various injection-based treatment methods (such as corticosteroid (CS), local anesthetic (LA), prolotherapy, platelet rich plasma (PRP), hyaluronic acid (HA), botulinum toxin injections (BTX) [4,5], and medical ozone gas injections) have gained popularity. In order to achieve this objective, a number of injection treatment approaches for different anatomical regions such as the intra-articular shoulder, the subacromial space, and the suprascapular nerve (SSN) have been described in the literature. In recent years, the combination of subacromial injection (SAI) with suprascapular nerve block (SSNB) has been used more frequently in order to reduce pain and enhance functional recovery in patients [6,7]. Additionally, recent studies have indicated that the combination of an SSNB with an SAI is superior in providing pain control for patients [8]. CSs are the preferred pharmacological agents for injections applied to different anatomical regions. However, the existing evidence for alternative injection therapies, such as PRP or HA, is inconclusive with respect to their efficacy in the short, medium, and long term, as well as the appropriate dosage and frequency of injections. There is no clear consensus in practice regarding these therapies [9]. The objective of this study was to compare the effects of SAI under ultrasound guidance alone versus SAI combined with SSNB on pain, function, and sleep quality in patients with shoulder pain unresponsive to conservative treatments due to RCL.

## 2. Materials and Methods

### 2.1. Study Design and Population

This prospective, observational study included 50 patients who presented to the hospital with unilateral shoulder pain between June 2022 and September 2022. The study protocol was approved by the local ethics committee (date: 8 June 2022, no: E-93471371-514.99) and conducted in accordance with the principles of the Declaration of Helsinki. The inclusion criteria of the study were age over 18 years, positive or negative results on examination of shoulder tests, diagnosis with RCLs by magnetic resonance imaging (MRI), symptoms persisting for more than three months, non-responsiveness to conservative treatment, and a visual analog scale (VAS) score of >4. The exclusion criteria of study were patients with a history of shoulder steroid injections, surgery, or fracture; pregnant and/or breastfeeding women; those with uncontrolled diabetes mellitus; malignancy; bleeding disorders; severe cardiopulmonary disease; sepsis; inflammatory arthritis and/or systemic infection; those with full-thickness tears in the shoulder rotator cuff muscles; a history of allergic reactions to the medications used; or skin infections at the injection site.

### 2.2. Interventions

The two techniques were explained to the patients and the procedures were subsequently performed on those who had consented to the treatment. The patients were equally divided into two groups: Group SAI, comprising patients who underwent a one-time local CS and LA injection into the subacromial area, and Group SAI + SSNB, including patients who underwent SSNB in addition to SAI.

SAI and SSNB procedures were performed as outpatient procedures by two experienced algology specialists, using standard monitoring (pulse oximetry, electrocardiography, and blood pressure) and ultrasound guidance (Toshiba Xario USDI-770A, Canon Medical System Corporation, Otawara, Japan), as described below.

After asepsis and antisepsis, the injection was performed using a 13 MHz linear ultrasound probe and a 22 G black-tipped needle with an in-plane approach. In this procedure, 40 mg (1 mL) triamcinolone and 80 mg (4 mL) of 2% prilocaine hydrochloride were injected into the subacromial bursae.

After asepsis and antisepsis, the injection was performed using a 13 MHz linear ultrasound probe and a 22 G, 8 cm needle with an in-plane approach. After visualizing the hypoechoic SSN beneath the transverse scapular ligament, 20 mg (1 mL) triamcinolone acetonide and 80 mg (4 mL) of 2% prilocaine hydrochloride were injected.

### 2.3. Outcomes

The efficacy of the triamcinolone acetonate application along with prilocaine hydrochloride to the subacromial region and SSN was evaluated using various parameters at the 30th minute, 1st week, 2nd week, 1st month, and 3rd month following the intervention. Demographic data, along with clinical characteristics and MRI findings prior to the intervention, were recorded for all patients. The following variables were used in the evaluation and follow-up.

The severity of shoulder pain was evaluated using both resting and moving VAS, 10-cm line, ranging from no pain (0) to maximum pain (10). VAS ≤ 4 was considered an adequate analgesia level. Analgesic consumption was as follows: in patients who were using non-steroidal anti-inflammatory drugs (NSAIDs) and/or opioids prior to the procedure, the impact of the treatment on drug utilization was evaluated through the administration of three questionnaires (1: drug use is similar, 2: drug use decreased, 3: drug use increased). Patients were instructed to take the analgesics they were using prior to the procedure in accordance with their VAS values (in cases where VAS was >4 or if the patient required it). The functional evaluation of the shoulder was developed by Roach et al. (1991), and its validity and reliability in Turkey was determined by Ciftci et al. (2021) with the shoulder pain and disability index (SPADI) [10,11]. Sleep quality was measured with the Pittsburgh Sleep Quality Index (PSQI) score, which was developed by Agargün et al. (1996) and whose validity and reliability study in Turkey was conducted by the same authors [12,13]. Patient satisfaction was evaluated at the conclusion of the study using a four-point scale (1: Poor, 2: Average, 3: Good, 4: Excellent). Evaluation of side effects was made as follows: any adverse effects or complications that may occur during or after the procedure were recorded.

### 2.4. Statistical Analysis

Data were analyzed using IBM SPSS version 26.0 software by SPSS Inc. in Chicago, IL, USA. To assess the demographic data, descriptive statistics (median, mean, standard deviation (SD), and minimum (min.) and maximum (max.) values) were calculated for each parameter for all groups. For comparisons between groups, normality of all parameters was determined using the Shapiro–Wilk test. The independent sample *t*-test was used to compare variables that were normally distributed, whereas the Mann–Whitney U test was used for non-normally distributed variables. The chi-square test was used to compare categorical data (clinical features). The normality of VAS, SPADI, PSQI, and satisfaction scores between groups was assessed using the Shapiro–Wilk test. In light of these findings, if at least one group was identified as not exhibiting a normal distribution, the Mann–Whitney U test was used for comparisons; if both groups were normally distributed, the independent sample *t*-test was utilized. The Friedman test was employed to ascertain the statistical significance of the observed changes in VAS, SPADI, PSQI, and satisfaction scores within the group at designated follow-up times, and the repeated measures were analyzed using the ANOVA test. The Wilcoxon signed-ranks test was used for post hoc comparisons of the Friedman test, while the Bonferroni test was used for the post hoc analysis of ANOVA for repeated measures. A chi-square test was used to ascertain whether there were significant differences in the rates of analgesic drug utilization between the various groups. All statistical analyses were conducted with a 95% confidence interval and a significance level of *p* < 0.05. Additionally, bivariate correlations were performed to assess the relationships between variables; specifically, Pearson’s correlation was applied for continuous variables, while Spearman’s rho correlations were used for ordinal variables. *p* < 0.05 was considered statistically significant. A cut-off for correlations was considered as follows: values between 0.3 and 0.7 indicated a moderate positive linear relationship, while values between −0.3 and −0.7 indicated a moderate negative linear relationship [14]. Since the number of data obtained because of comparing the correlation values was 128, it was written in the Section 3 by specifying the minimum and maximum values, rather than as separate tables.

A power analysis was conducted using the Power and Sample Size program. For the primary variable, VAS scores, a significant decrease of 4 points or more was considered meaningful with a 95% confidence interval and 80% power. This analysis indicated that a minimum of 22 patients per group was necessary. To account for potential follow-up/data loss, 25 patients were included in each group [8,15].

## 3. Results

**Figure 1:**
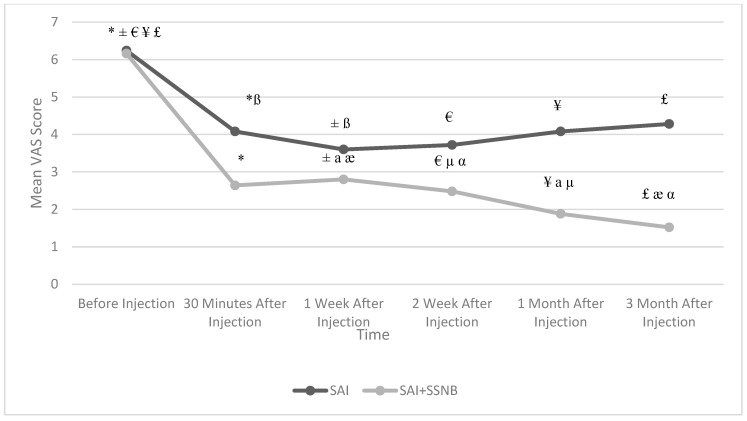
Change in mean resting VAS scores in both groups according to follow-up time. SAI: Subacromial Injection, SSNB: Suprascapular Nerve Block, VAS: Visual Analog Scale. * Before Injection vs. 30th min. (*p* < 0.01), ± Before Injection vs. 1st week (*p* < 0.01), € Before Injection vs. 2nd week (*p* < 0.01), ¥ Before Injection vs. 1st month (*p* < 0.01), £ Before Injection vs. 3rd month (*p* < 0.01), ß 30th min. vs. 1st week (*p* < 0.05), a 1st week vs. 1st month (*p* < 0.05), æ 1st week vs. 3rd month (*p* < 0.01), µ 2nd month vs. 1st month (*p* < 0.05), α 2nd week vs. 3rd month (*p* < 0.01).

**Figure 2:**
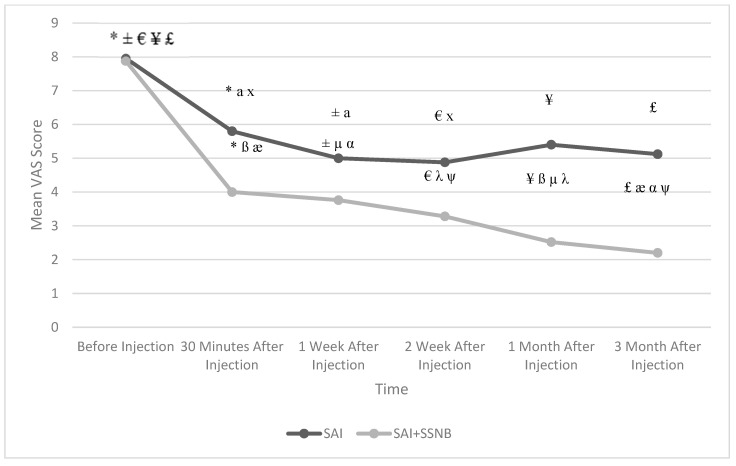
Change in mean moving VAS scores in both groups according to follow-up time. SAI: Subacromial Injection, SSNB: Suprascapular Nerve Block, VAS: Visual Analog Scale * Before Injection vs. 30th min. (*p* < 0.01), ± Before Injection vs. 1st week (*p* < 0.01), € Before Injection vs. 2nd week (*p* < 0.01), ¥ Before Injection vs. 1st month (*p* < 0.01), £ Before Injection vs. 3rd month (*p* < 0.01), a 30th min. vs. 1st week (*p* < 0.05), x 30th min. vs. 2nd week (*p* < 0.05), ß 30th min. vs. 1st month (*p* < 0.01), æ 30th min. vs. 3rd month (*p* < 0.05), µ 1st week vs. 1st month (*p* < 0.01), α 1st week vs. 3rd month (*p* < 0.01), λ 2nd week vs. 1st month (*p* < 0.05), ψ 2nd week vs. 3rd month (*p* < 0.01).

The mean ages of the patients were 49.52 ± 9.7 and 57.48 ± 11.6 years in the SAI group and SAI + SSNB group, respectively. Their baseline demographic data and clinical characteristics are shown in Table 1. There was no statistically significant difference between the groups in terms of resting and moving VAS scores before treatment (*p* = 0.804 for resting; *p* = 0.945 for moving; Table 2). There were statistically significant differences between two groups of resting and moving VAS scores (*p* < 0.05; Table 2), except for the 1st week resting VAS score (*p* = 0.076; Table 2). Compared to the mean resting and moving VAS scores before treatment in both groups, the mean resting and moving VAS scores at all follow-up times were found to be statistically significantly lower (*p* < 0.05; Figure 1 for resting; Figure 2 for moving). However, it was observed that the adequate level of analgesia targeted for pain control in the SAI group (VAS ≤ 4) was not achieved at the 1st and 3rd months. In the SAI + SSNB group, both resting and moving VAS scores decreased significantly more than baseline compared to the SAI group (*p* < 0.05).

**Figure 3:**
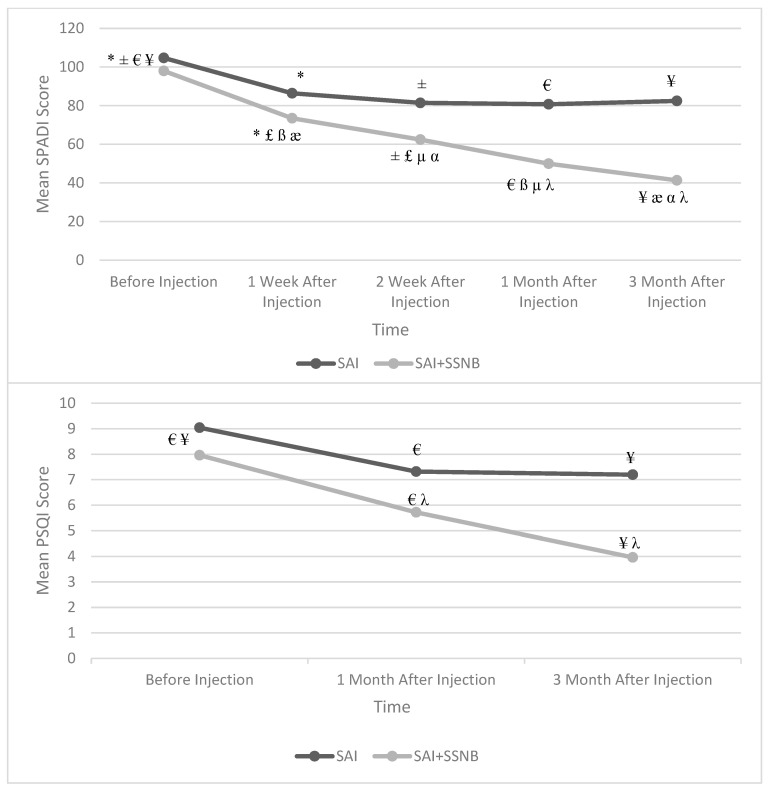
Change in mean “SPADI” and “PSQI” scores according to follow-up time in both groups. SPADI: Shoulder Pain and Disability Index, PSQI: Pittsburgh Sleep Quality Index, SAI: Subacromial Injection, SSNB: Suprascapular Nerve Block. * Before Injection vs. 1st week (*p* < 0.01), ± Before Injection vs. 2nd week (*p* < 0.01), € Before Injection vs. 1st week (*p* < 0.01), ¥ Before Injection vs. 3rd month (*p* < 0.01), £ 1st week vs. 2nd week (*p* < 0.01), ß 1st week vs. 1st month (*p* < 0.01), æ 1st week vs. 3rd month (*p* < 0.01), µ 2nd week vs. 1st month (*p* < 0.01), α 2nd week vs. 3rd month (*p* < 0.01), λ 1st month vs. 3rd month (*p* < 0.01).

There was no statistically significant difference between the groups in the mean pre-treatment SPADI scores (*p* = 0.353). Compared to the pre-treatment mean SPADI scores in both groups, the mean SPADI scores at all follow-up times were significantly lower (*p* < 0.05; Figure 3). There was no statistically significant difference between groups in the % change in mean SPADI scores from baseline, although a greater decrease was observed in the SAI + SSNB group (*p* = 0.094).

There was no statistically significant difference between the groups in terms of pre-treatment PSQI scores (*p* = 0249). Compared with the mean pre-treatment PSQI scores in both groups, the mean PSQI scores at all follow-up times were significantly lower (*p* < 0.05; Figure 3). When comparing the percentage change in mean PSQI scores, there was no statistically significant difference between the groups (*p* = 0.459), although there was a greater decrease in the SAI + SSNB group.

**Figure 4:**
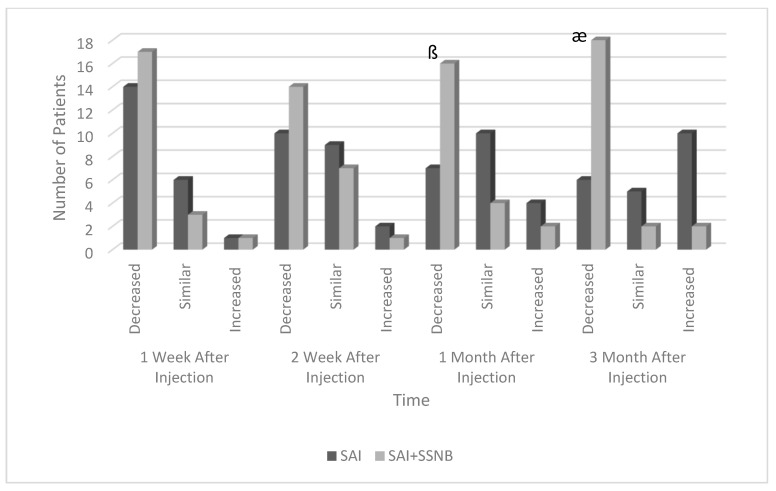
Change in analgesic drug use of patients according to follow-up time in both groups. SAI: Subacromial Injection, SSNB: Suprascapular Nerve Block. ß the difference in the first month after injection is statistically significant (*p* < 0.05), æ the difference in the third month after injection is statistically significant (*p* < 0.01).

Rates of analgesic use in both groups are shown in Figure 4. There was no statistically significant difference between the groups in the change in analgesic use rates at the 1st and 2nd weeks. There was a statistically significant difference between the groups in the rate of change in analgesic use at 1st and 3rd months. Similarly, the rate of those with increased pain was found to be significantly higher in the SAI group than in the SAI + SSNB group (Figure 4). A statistically significant difference was found between the groups in the amount of change in satisfaction scores at all follow-up times throughout the follow-up interval (*p* < 0.01). Compared to the SAI group, patient satisfaction scores were statistically significantly higher in the SAI + SSNB group.

No serious side effects or complications were observed during or after treatment. Additionally, no increase in HbA1c levels was observed after treatment in either group. There was a statistically significant and positive correlation between the resting and moving VAS scores and the SPADI scores (*p* < 0.00001–0.001) (R = 0.6–0.89). In addition, there was a statistically significant positive correlation between resting and moving VAS scores and PSQI scores (*p* < 0.00001–0.21) (R = 0.25–0.83). On the other hand, a statistically significant negative correlation was found between resting and moving VAS scores and patient satisfaction (*p* < 0.00001–0.88) (R = 0.05–0.80). There was also a statistically significant negative correlation between SPADI and PSQI scores and patient satisfaction (*p* = 0.0008–0.87) (R = 0.03–0.62).

## 4. Discussion

In this study, isolated SAI and the addition of SSNB therapies used for the treatment of RCLs were compared. It was suggested that both types of treatment provide positive results on RLCs; however, the targeted sufficient analgesia level for pain control during movement was not achieved with isolated SAI. The combination of SSNB and SAI seemed more effective at the end of the third month.

The origin of shoulder pain is multiple and can involve muscle and tendons of the rotator cuff as well as bones and ligaments of joints [16]. RCLs are the most common causes of shoulder pain [17]. The process, which usually begins as tendinopathy or partial tears, can progress to full-thickness tears or adhesive capsulitis, and at times requires surgical therapy and subsequent rehabilitation [18,19]. Oral analgesics, physiotherapy and exercise, intra-articular injections, and peripheral nerve blocks are common treatment approaches [16]. There is no consensus on which approach is superior and no accepted algorithm. In the study by Fawcett et al. [20], the application of combined CS and LA to the subacromial and subdeltoid bursae in 376 patients with shoulder pain resulted in significant improvement in shoulder pain and function in two thirds of the patients. Significant improvement was observed in a quarter of patients in the long term. In a meta-analysis by Arroll et al. that compared the efficacy of subacromial and intra-articular CS injections with physiotherapy, placebo, and NSAIDs, subacromial CS injections were shown to be more effective than NSAIDs and physiotherapy techniques at nine months’ follow-up, and it was reported that higher doses may also be more effective. The study by Buchbinder et al. [21] found that CS and SAI in RCLs may have short-term and limited effects. Although CS has partial beneficial effects in the short term, the potential side effects limit using repeated CS injections in subacromial region. The study by Puzzitiello et al. [22] reported that CS reduced cell proliferation, changed collagen and extracellular matrix composition, inhibited inflammatory pathways, increased adipocyte differentiation, decreased cell viability, and increased apoptosis. These changes began within 24 h of injection and persisted for 2–3 weeks [23,24,25]. These findings support the limitation of CS injections to a maximum of three per year.

Recent studies have indicated an increased importance of SSNB in shoulder pain due to different etiologies [26,27]. SSNs sensory branches receive 70% of their sensation from the shoulder. Various treatment approaches have been described, such as LA and/or CS injection and radiofrequency ablation for SSN [28,29,30,31]. It is seen that there is no standard application in the current literature. In the study by Lee et al. [32], isolated suprascapular and subscapular nerve blocks were applied to 52 patients with shoulder pain, and it was observed that the patients’ pain decreased, and their functionality increased. The authors reported that the method can be safely used for shoulder pain regardless of etiology. The study by Bae et al. [33] compared the effects of SAI and SSNB in 60 patients with shoulder pain and showed that SSNB provided better pain control than SAI at four weeks. Conversely, the mechanisms of SSNB are still uncertain, and there is no optimal method that has been identified for SSNB. A systematic review was conducted by Smith et al. [34] to ascertain the current methods and drugs employed for SSNB in the nonsurgical treatment of adults with chronic shoulder pain. A total of 21 different combinations were used, with bupivacaine and methylprednisolone being most common. Although there is evidence in the literature supporting the addition of CSs to LA on the grounds that they enhance the effect of the injection by prolonging the duration of the nerve block, the argument whether this improves outcomes remains unclear currently [35].

The combination provides effective, simple, reliable, and inexpensive treatment options for RCLs. The study by Atalay et al. [36] showed that adding SSNB to intra-articular CS injection in patients with shoulder pain due to adhesive capsulitis provided rapid pain relief and improved function. The study by Jung et al. [37] showed that intra-articular CS injection with SSNB was more effective than other treatments for partial rotator cuff tears. The authors stated that afferent nociceptive stimuli from the shoulder joint were blocked by the effect of steroids used in suprascapular nerve blockade, and this reduced pain complaints. Further studies are required to investigate the efficacy of combining SSNB with other interventions. Some studies have demonstrated that combining SSNB and SAI can facilitate superior pain control and accelerated functional recovery [8]. In the study conducted by Yılmaz [8], the effects of a subacromial (CS) injection and the addition of SSNB were evaluated in 66 patients with SIS, and it was found that this combination provided better results on pain and functionality. In contrast to the methodology of Yılmaz’s study, our study used prilocaine hydrochloride, and 40 mg CS injection instead of 20 mg in SAI. In addition, 20 mg of CS was used with LA during SSNB in our study. Despite the higher CS dose, there was no observed increase in HbA1c levels in either group. Further studies are required to determine the optimal dosage for pain, functionality, and sleep quality.

Early interventions are important to prevent pain associated with RCLs from impeding joint range of motion. In cases where pain is particularly severe, interventions should be considered before exercise and physical therapy applications to preserve shoulder functions. In recent literature, there are studies in which shoulder functions are evaluated using different tests, in addition to pain control. In the study by Yılmaz [8], isolated subacromial CS and LA was applied to one group of 66 patients with SIS, and SSNB was added in the other group. Patients’ functionality was monitored using the Disabilities of the Arm, Shoulder, and Hand (DASH) score. The decrease in score was less pronounced in the group that received an isolated subacromial CS and LA injection than in the group that also received SSNB. A review of shoulder pathologies revealed that superior improvement in shoulder function is achieved when SSNB is combined with intra-articular or SAI. Our study demonstrated that patients who received SSNB and SAI together exhibited superior improvement in shoulder function. Additionally, a significant negative correlation was identified between patient satisfaction scores and SPADI scores. In this study, isolated SAI and the addition of SSNB therapies used for the treatment of RCLs were compared. It was suggested that both types of treatment provide positive results on RLCs; however, the targeted sufficient analgesia level for pain control during movement was not achieved with isolated SAI. The combination of SSNB and SAI seemed more effective at the end of the third month. It is of great importance to emphasize the significance of injection techniques and ultrasound-guided interventions in the context of rehabilitation processes, particularly in the treatment of shoulder diseases. A review of the literature reveals that these approaches have been demonstrated to accelerate the achievement of rehabilitation outcomes, with efficacy in enhancing muscle activity and joint range of motion. In a study conducted by Farì et al. [38], it was demonstrated that real-time monitoring of muscle activity and optimizing joint range of motion during shoulder pain rehabilitation facilitated enhanced rehabilitation outcomes. This has made a significant contribution, particularly in the context of wheelchair basketball. Similarly, in another study, it was stated that increasing supraspinatus muscle activity could accelerate the rehabilitation process for shoulder pain [39]. It was concluded that such methods could enable patients to return to their daily activities more quickly and make the treatment process more effective.

Another important parameter evaluated in our study is sleep quality. Although the strong relationship between pain and sleep is known, there are not many studies on this subject [40]. Tekeoglu et al. [41] conducted a study in which they compared 40 patients with chronic shoulder pain due to SIS and 43 healthy individuals. They determined that the group with chronic shoulder pain exhibited higher PSQI scores. Mulligan et al. [42] examined 343 patients with shoulder pain in their study and compared the patients’ functionality, pain, and sleep quality. Even in cases of the SIS that had the least disruptive effect on sleep quality, the average PSQI score was 8.59. The highest mean PSQI score was 12.07 in cases of adhesive capsulitis. In our study, superior improvement in sleep quality was observed in patients who underwent SAI and SSNB together. Furthermore, a significant negative correlation was identified between patient satisfaction scores and PSQI scores.

### 4.1. Limitations

The absence of a control group and an isolated SSNB group, in addition to a short follow-up period, are potential limitations of this study. The fact that the patients did not have a standard, professional physical therapy and exercise program after the procedures can also be seen as a shortcoming of this study. Moreover, with the advancement of artificial intelligence and machine learning methods, future studies should explore this aspect, especially in physical and rehabilitation medicine and tendinopathies, as in previously developed studies [43]. We believe our study will pioneer prospective, randomized controlled and long-term follow-up studies comparing different types and doses of injection agents.

### 4.2. Conclusions

In conclusion, the combination of SAI and SSNB instead of SAI in shoulder pain due to RCLs unresponsive to conservative treatments represents a method that reduces the use of analgesic drugs and VAS scores in the short to medium term, increases functional recovery and sleep quality, has high patient satisfaction and effectiveness, and has a low potential for side effects. In our study, as in other few similar studies in the literature, we can conclude that the application of both SAI and SSNB yielded superior results. Further research is required to establish a standard for SSNB application with a view to providing guidance for future clinical applications.

## Figures and Tables

**Table 1:** Demographic and clinical characteristics of patients.

	SAI (*n* = 25)	SAI + SSNB (*n* = 25)	*p*
Age	49.52 ± 9.7	57.48 ± 11.6	0.011 *
Length (meter)	1.64 ± 0.1	1.62 ± 0.1	0.411
Weight (kg)	77.6 ± 10.2	81.04 ± 13.2	0.308
Body Mass Index (BMI)	29.1 ± 4.3	30.99 ± 5.1	0.159
Gender			
Male	8 (32%)	4 (16%)	0.321
Female	17 (68%)	21 (84%)	
Dominant Hand			
Left	2 (8%)	2 (8%)	1
Right	23 (92%)	23 (92%)	
Affected Hand			
Left	8 (32%)	7 (28%)	0.758
Right	17 (68%)	18 (72%)	
Profession			
Worker	8 (32%)	3 (12%)	0.08
Officer	5 (20%)	2 (8%)	
Housewife	12 (48%)	18 (72%)	
Retired	0 (0%)	2 (8%)	
Diabetes Mellitus			
No	20 (80%)	14 (56%)	0.128
Yes	5 (20%)	11 (44%)	
Hypertension			
No	17 (68%)	12 (48%)	0.252
Yes	8 (32%)	13 (52%)	
Hyperlipidemia			
No	22 (88%)	22 (88%)	1
Yes	3 (12%)	3 (12%)	
Coronary Artery Disease			
No	25 (100%)	20 (80%)	0.018 *
Yes	0 (0%)	5 (20%)	
Asthma			
No	25 (100%)	21 (84%)	0.037 *
Yes	0 (0%)	4 (16%)	
Hypothyroidism			
No	22 (88%)	20 (80%)	0.44
Yes	3 (12%)	5 (20%)	
Other Diseases			
No	22 (88%)	21 (84%)	0.684
Yes	3 (12%)	4 (16%)	
Patients’ MRI Findings			
Partial Tear	11	15	0.222
Tendinosis	11	7	
Partial Fear + Tendinosis	3	3	
NEER test			
Negative	4 (16%)	4 (16%)	1
Positive	21 (84%)	21 (84%)	
HAWKINS test			
Negative	5 (20%)	3 (12%)	0.44
Positive	20 (80%)	22 (88%)	
JOBE test			
Negative	13 (52%)	19 (76%)	0.14
Positive	12 (48%)	6 (24%)	
SPEED test			
Negative	21 (84%)	16 (64%)	0.196
Positive	4 (16%)	9 (36%)	
Range of Motion			
Restricted	4 (16%)	6 (24%)	0.725
Not Restricted	21 (84%)	19 (76%)	
Range of Motion			
Painless	4 (16%)	3 (12%)	0.684
Painful	21 (84%)	22 (88%)	
Previous Treatments NSAIDs			
No	4 (16%)	2 (8%)	0.384
Yes	21 (84%)	23 (92%)	
Previous Treatments OPIOID			
No	21 (84%)	18 (72%)	0.496
Yes	4 (16%)	7 (28%)	
Pre-treatment Hba1c Levels	6.03 ± 0.4	6.64 ± 0.7	0.062
Use of Analgesics Before Injection			
No	5 (20.0%)	4 (16.0%)	0.713
Yes	20 (80.0%)	21 (84.0%)	

Values are given as average ± or number (%). *: *p* < 0.05 statistically difference between groups. SAI: Subacromial Injection, SSNB: Suprascapular Nerve Block.

**Table 2:** Descriptive characteristics of patients’ resting and moving VAS scores by follow-up time.

Resting VAS	SAI (*n* = 25)			SAI + SSNB (*n* = 25)			*p*
	Median	Min.–Max.	Mean ± SS	Median	Min.–Max.	Mean ± SS	
Before Injection	6	4–9	6.24 ± 1.5	6	4–10	6.16 ± 1.7	0.804
30 Min After Injection	4	2–7	4.08 ± 1.6	2	0–8	2.64 ± 1.8	0.003 **
1 Week After Injection	3	2–8	3.6 ± 1.5	3	0–6	2.8 ± 1.3	0.076
2 Weeks After Injection	3	1–8	3.72 ± 1.7	2	0–6	2.48 ± 1.3	0.006 **
1 Month After Injection	4	1–7	4.08 ± 1.8	2	0–7	1.88 ± 1.6	0.0001 **
3 Months After Injection	4	1–8	4.28 ± 2.4	1	0–7	1.52 ± 2	0.0001 **
**Moving VAS**		**SAI** **(*n* = 25)**			**SAI + SSNB (*n* = 25)**		***p***
	**Median**	**Min.–Max.**	**Mean ± SS**	**Median**	**Min.–Max.**	**Mean ± SS**	
Before Injection	8	5–10	7.96 ± 1.4	8	4–10	7.88 ± 1.7	0.945
30 Min After Injection	6	2–9	5.8 ± 1.8	4	0–10	4 ± 2.2	0.003 **
1 Week After Injection	4	3–9	5 ± 1.8	4	0–7	3.76	0.019 *
2 Weeks After Injection	4	2–10	4.88 ± 2.1	3	1–7	3.28 ± 1.7	0.005 **
1 Month After Injection	5	2–10	5.4 ± 2.4	2	0–8	2.52 ± 2.1	0.0001 **
3 Months After Injection	6	1–9	5.12 ± 2.9	1	0–9	2.2 ± 2.5	0.0001 **

*: *p* < 0.05; **: *p* < 0.01. SAI: Subacromial Injection, SSNB: Suprascapular Nerve Block, VAS: Visual Analog Scale.

## Data Availability

The raw data supporting the conclusions of this article will be made available by the authors on request.
